# The Treatment of Activated PI3Kδ Syndrome

**DOI:** 10.3389/fimmu.2018.02043

**Published:** 2018-09-07

**Authors:** Tanya I. Coulter, Andrew J. Cant

**Affiliations:** ^1^Regional Immunology Service, Belfast Health and Social Care Trust Belfast, United Kingdom; ^2^Department of Paediatric Immunology and Stem Cell Transplant Unit, Newcastle upon Tyne Hospitals NHS Foundation Trust Newcastle upon Tyne, United Kingdom

**Keywords:** activated PI3 kinase delta syndrome, PASLI, HSCT, primary immunodeficiency, APDs

## Abstract

Activated phosphoinositide 3-kinase δ syndrome (APDS), also known as PASLI disease (p110d-activating mutation causing senescent T cells, lymphadenopathy, and immunodeficiency) are combined immunodeficiencies resulting from gain-of-function mutations in the genes (*PIK3CD* and *PIK3R1*) encoding the subunits of phosphoinositide 3-kinase δ (PI3Kδ) and were first described in 2013. These mutations result in the hyperactivation of the PI3K/AKT/mTOR/S6K signally pathways. In this mini-review we have detailed the current treatment options for APDS. These treatments including conventional immunodeficiency therapies such as immunoglobulin replacement, antibiotic prophylaxis, and hematopoietic stem cell transplant. We also discuss the more targeted therapies of mTOR inhibition with sirolimus and selective PI3Kδ inhibitors.

Activated phosphoinositide 3-kinase δ syndrome (APDS), also known as PASLI disease (p110d-activating mutation causing senescent T cells, lymphadenopathy, and immunodeficiency) is a combined immunodeficiency resulting from dominant, gain-of-function mutations in the genes encoding p110δ (*PIK3CD*) and p85α (*PIK3R1*), the catalytic and regulatory subunits of phosphoinositide 3-kinase δ (PI3Kδ). These mutations result in the hyperactivation of the PI3K/AKT/mTOR/S6K signally pathways in immune cells (1–3).

Patients with APDS may develop immunodeficient and immunodysregulatory features including recurrent respiratory tract infections, bronchiectasis, herpesvirus infections, autoimmunity, non-neoplastic lymphoproliferation, and lymphoma, as well as neurodevelopment delay and growth retardation (4, 5). Clinical cohort studies show that the manifestations of APDS are highly variable, even within families carrying the same mutation, ranging from asymptomatic adult patients to those with primary antibody deficiency, those with a profound immunodeficiency causing early death, to others suffering from lymphoproliferation and malignancy. Thus, the treatment of patients with APDS has varied considerably from simple observation to haematopoietic stem cell transplant (HSCT) in childhood (4, 5). These studies also suggest that disease manifestations mostly have a pediatric onset with recurrent respiratory infections usually the first manifestation occurring in infancy or childhood [0–10 years of age in (4, 5)] followed by bronchiectasis and autoimmunity in later childhood (4–6).

APDS due to gain-of-function mutations in the genes encoding p110δ (*PIK3CD*) and p85α (*PIK3R1*) are termed APDS1 and APDS2 respectively. Previously we have described a cohort of 53 patients with APDS1, 50 of whom had the E1021K mutation and 3 of with the E525 mutation (4). Similarly, Elkaim et al. (5) studied a cohort of 36 patients with APDS2 due to mutations within splice acceptor and donor sites in exon 11 of *PI3KR1*. Comparing the clinical features of these APDS1 and APDS2 cohorts suggests a similar phenotype with the exception that the APDS1 cohort had a higher prevalence of bronchiectasis (60 vs. 18%) and lower prevalence of lymphoma (13 vs. 25%) compared to APDS2, growth retardation was only reported in the APDS2 cohort and there was an asymptomatic E1021K mutation carrier in the APDS1 cohort compared no asymptomatic individuals in the APDS2 cohort. To date there has been no evidence that the different *PIK3CD* and *PIK3R1* genes mutations lead to different APDS1 and APDS2 phenotypes, however this does require further study. Also, there has been no suggestion to date that APDS1 and APDS2 should be treated differently.

Although APDS was only first described in 2013, many individuals were already under the care of immunology services with diagnoses such as common variable Immunodeficiency (CVID) or combined immunodeficiency (CID), and being treated with conventional therapies including prophylactic antibiotics, immunoglobulin replacement, and in some cases HSCT (1, 3). As patients with APDS can also develop autoimmune and inflammatory complications, others were on various immunosuppressive therapies. Our increasing understanding of the underlying mechanism of APDS however suggests a role for more targeted treatment in this disorder such as direct inhibition of the activated PI3Kδ by selective PI3Kδ inhibitors or inhibition of the downstream mTOR pathway by Sirolimus (3, 7).

In this mini-review will we review how APDS is treated, examining the experience of using conventional immune deficiency therapies in APDS including HSCT, and the more recent experience of selective PI3Kδ inhibitors and Sirolimus. These treatment options are summarized in Table 1.

**Table 1:** Treatment options in Activated PI3Kδ Syndrome (APDS).

**Treatment**	**Benefit in APDS**	**Proposed mechanism of action in APDS**
Antimicrobial prophylaxis (e.g., Trimethoprim/Sulfamethoxazole or Azithromycin)	Reduction in respiratory tract infections	Prevention of respiratory bacterial infections
Immunoglobulin replacement therapy	Reduction in respiratory tract infections	Correction of antibody deficiency secondary to APDS
Haematopoietic stem cell transplantation	Reduction in respiratory & herpes infections Reduction in lymphoproliferation & autoimmunity	Replacement leukocytes effected by PI3Kδ hyperactivation
Sirolimus (Rapmycin)	Reduction in lymphoproliferation	Reduction in mTOR hyperactivation
Selective PI3Kδ inhibitors (e.g., Leniolisib)	Reduction in lymphoproliferation	Reduction in PI3Kδ hyperactivation

## Conventional PID treatments in APDS

Individuals with APDS often have demonstrable antibody deficiency and lymphopenia and recurrent infections. These complications have led immunologists to treat APDS with standard supportive therapies such as prophylactic antibiotic therapy, immunoglobulin replacement therapy (IRT), and HSCT. (4) and (5) revealed the frequency of conventional treatments in patients with APDS.

## Antimicrobial prophylaxis

In our APDS1 cohort 62% of patients currently received and 9% had previously received antibiotic prophylaxis. Similarly, Elkaim et al. (5) found 61% of patients with APDS2 were on antibiotic prophylaxis. The antibiotic prophylactic regimens used were similar to those used in patients with antibody defects, largely Trimethoprim/Sulfamethoxazole or Azithromycin. Antibiotic prophylaxis alone sufficed for some patients, however most needed IRT (4, 5). Combined IRT and antibiotic prophylaxis is especially important for patients with respiratory tract infections in the context of established bronchiectasis (4, 5).

Although APDS is also associated with persistent, severe, or recurrent herpesvirus infections (3, 4), in our cohort of patients with APDS1 only 6% were on long term Acyclovir/Valaciclovir due to previous herpes infections despite 49% of patients having had a significant herpes infection previously. 13% of the patients in our APDS1 cohort received antifungal prophylaxis due to previous mucocutaneous candidiasis. No cases of Aspergillus species infection in APDS have been reported.

In individuals with APDS who have received *Mycobacterium bovis* bacillus Calmette Guèrin (BCG) vaccination, persistent local BCG site skin reactions have been reported (4, 5). Chiriaco et al. (8) reported that APDS patient monocytes-derived macrophages failed to eliminate BCG infection *in vitro*, raising the question as to whether APDS patients should be given anti BCG therapy. However, disseminated BCGosis and other mycobacterial infections have not been reported in APDS, and so whilst individual patients have been treated for local BCG infections, APDS patients are not routinely placed on anti-mycobacterial prophylaxis.

## Immunoglobulin replacement therapy

Many patients with APDS have antibody deficiency and associated recurrent respiratory tract infections. These antibody defects range from poor vaccine responses and IgG subclass deficiencies to significantly reduced IgG and IgA with normal or elevated IgM. Many patients with APDS have a historical diagnosis of specific antibody deficiency, subclass deficiency, common variable immune deficiency and hyper IgM syndrome and have been treated with IRT.

The majority of APDS patients, 87% in the Coulter et al. (4) APDS1 cohort, and 89% in the Elkaim et al. (5) APDS2 cohort received long-term IRT with a reduction in bacterial infection burden in most cases. IRT was administered as in other antibody deficiencies, i.e., as a long-term therapy, given as intravenous (IVIG) or subcutaneous (SCIG) infusions. IRT is usually commenced at a dose of 0.4 g/kg/month in antibody deficient patients without bronchiectasis and at a higher dose of 0.6 g/kg/month in patients with bronchiectasis (9–11).

IRT is of reported benefit in many patients with APDS reducing respiratory tract infections (4, 12, 13). However, IRT does not appear to prevent the development of the herpes infections nor lymphoproliferative, autoimmune/inflammatory complications nor lymphoma (4, 12, 14, 15). Furthermore, in some APDS patients bronchiectasis has progressed despite optimal IRT (4, 5, 12, 15).

Many patients with APDS develop recurrent respiratory tract infections which often progress rapidly to bronchiectasis in childhood (4, 5). Individuals with APDS and bronchiectasis should be treated as in other patients with bronchiectasis, including airway clearance, physiotherapy, influenza immunization, patient education antibiotic treatment for infective exacerbations, the consideration of interval intensive physiotherapy and antibiotics, nebulized hypertonic saline and bronchodilators, and prophylactic antibiotics where these therapies may benefit. It is recommended that the management and monitoring of patients with bronchiectasis and immune deficiency should be provided through a joint clinical immunology and respiratory (±pediatricians) care model with access to specialist respiratory nursing and physiotherapy services with an expertise in bronchiectasis (16–18).

## Haematopoietic stem cell transplantation

APDS can present as or evolve into a profound combined immunodeficiency which leads into significant morbidity and early death. Allogenic haematopoietic stem cell transplantation (HSCT) has been performed to treat life-threatening infections and as part of treatment for lymphoma (4, 5, 19). Nademi et al. (19) reported a series of eleven patients with APDS and severe immune deficiency who underwent HSCT in seven pediatric centers. The diagnosis of APDS was made prior to HSCT in five patients and retrospectively in six. Ten of the 11 bore the E102IK *PIK3CD* mutation, and one a *PIK3R1* mutation. All had suffered severe sinopulmonary infection, four had bronchiectasis, six severe Herpes family virus infection, four enteropathy/colitis, eight lymphoproliferation, one intestinal obstruction, three significant progressive liver disease due to Cryptosporidium infection, one had glomerulonephritis and one glomerulosclerosis. Five had received steroids ± Sirolimus and seven immunoglobulin, and all were deteriorating despite their treatment. Age at transplant ranged from 5 to 23 years. Peripheral blood stem cells were used in 7 of the 11, five were transplanted from a matched unrelated donor, four from a matched sibling and two from a mismatched donor. Various conditioning regimes were used, mostly Fludarabine with either Treosulphan or Melphalan, and all but two were given serotherapy. Two patients died from CMV/Adenovirus pneumonitis and idiopathic pneumonitis respectively. Mild (Grade I or II) Graft vs. Host Disease occurred in nine patients, but fully resolved in all. Two patients had autoimmune haemolytic anemia, and one encephalitis, these complications also fully resolving. Eight of the nine surviving patients are off IVIG and well, one has got low donor chimerism and is awaiting a second HSCT (19). These preliminary data show that HSCT can be curative with resolution of pre HSCT signs and symptoms, and survival looks to be similar to that seen after HSCT for other PIDs. Indeed, considering the considerable pre HSCT morbidity in this cohort, less success might have been expected, and so it seems a promising option for patients with progressive disease unresponsive to other treatment. To date, long term sequelae do not seem to be a problem with follow up between 8 months and 16 years. However, the risks and benefits of HSCT in patients with severe lung disease are not clear, and longer term follow up in a larger cohort is needed to ascertain the place of HSCT in treating APDS.

## Immunosuppression

Thirty-four percent of our APDS1 cohort had autoimmune or inflammatory disease with 30% of the cohort treated with at least one course of immunosuppressive therapy. Cytopenias were the most common autoimmune disease in APDS, but conditions described include renal disease, inflammatory colitis, exocrine pancreatic insufficiency, seronegative arthritis, cirrhosis, and sclerosing cholangitis. In some cases renal or liver transplantation was necessary (4, 5).

The autoimmune cytopenias have been found to be responsive to steroids, rituximab and splenectomy (4, 12). Rituximab therapy has been noted to be complicated by sustained B-cell lymphopenia (4). Elgizouli et al. (12) described good clinical response from prednisolone and maintenance mesalazine in inflammatory bowel disease in APDS1. Hartman et al. (20) described two patients with APDS and primary sclerosing cholangitis who underwent liver transplantation. In one case the cholangitis relapsed 3 years after the original transplant, and the patient was awaiting re-transplantation.

Non-neoplastic lymphoproliferation including lymphadenopathy, splenomegaly, hepatomegaly with lymphoid aggregates in the respiratory and gastroenteric tract being a very frequent finding in APDS. We found Rituximab to be of some benefit in the management of these non-neoplastic lymphoproliferation in five patients with APDS1 (4).

## Sirolimus

The mTOR inhibitor, Sirolimus (Rapamycin) has been found to decrease in non-neoplastic lymphoproliferation (3–5, 21, 22). mTOR (mammalian target of rapamycin) is activated downstream of PI3K and has a prominent role in T cell metabolism and the regulation of immune responses (2, 3). Previously Sirolimus therapy had been reported in a case of APDS to reduce hepatosplenomegaly and lymphadenopathy, increase naïve T cell frequencies, and restore T cell proliferation and IL-2 secretion (3). Recently Maccari et al. (6) published the initial findings of the ESID APDS registry. They looked specifically at the use of Sirolimus/Rapamycin in APDS and noted a significant benefit in the treatment of non-neoplastic lymphoproliferative disease with 8/25 having complete response and 11/25 having partial response to sirolimus. Sirolimus was found to be of less benefit in treating APDS related-cytopenias and gastrointestinal disease with complete response in 3/14 and 3/15 cases and a partial response in 2/14 and 3/15 of cases respectively. Thus, studies to date support the use of Sirolimus in treating APDS-related non-neoplastic lymphoproliferation, though some patients may only develop partial benefit and lymphoproliferation may reoccur after treatment cession. The long-term benefits and risks of Sirolimus therapy remain to be determined.

## Selective PI3Kδ inhibitors

Selective PI3Kδ inhibitors have the potential to offer a targeted treatment option for APDS patients with greater efficacy and fewer side effects. PI3K inhibitor therapies have been tested in oncology trials, resulting in the approval of Idelalisib, for treatment of chronic lymphocytic leukemia and non-Hodgkin lymphoma (23, 24). However, Idelalisib has a considerable side-effect profile, including pneumonitis, transaminitis, and colitis (24).

Leniolisib (CDZ173), is a potent oral inhibitor of the p110δ subunit of PI3Kδ which is currently being studied for the treatment of APDS by Novartis (NCT02435173) (7, 25). Rao et al. (7) described a 12-week, open label, multisite clinical trial involving six individuals with APDS, all with lymphadenopathy and splenomegaly on CT/MRI at baseline. After an initial screening period of up to 50 days, including an immunosuppressive/immunomodulatory treatment wash-out period, all patients received Leniolisib in escalating doses (10, 30, 70 mg BD for 4 weeks each receiving 12 weeks total). After the 12 weeks of Leniolisib treatment, significant reduction in lymph node sizes (mean 40%, 13–65%) and spleen volume (mean 39%, range 26–57%) was seen in all patients. Patients reported an increase in wellbeing (Patient global assessment questionnaire 11 ± 11 mm), decrease in fatigue and less disease activity, as assessed by the physician global assessment questionnaire. There was also an improvement in immunological markers with a normalization of transitional B cell and naive B cell populations, and reduction of senescent (CD57+ CD4-) T cells, PD-1+CD4+ T cells, IgM levels and the cytokines and chemokines TNF, IFNγ, CXCL13 & CXCL10. Five of six patients proceeded to enroll in the open-label long term extension study using Leniolisib 70 mg BD. The sixth patient reportedly did not enroll due to logistical reasons related to traveling. Rao et al. (7) reported that during the first 9 months of this extension study no patient has experienced significant adverse effects. This initial report supports that Leniolisib, like Sirolimus, may be a treatment for APDS-related lymphoproliferation. However, the improvements in patient well-being and immunological parameters also suggests Leniolisib and other PI3Kδ inhibitors could have wider benefits for individuals with APDS, athough further studies are required, particularly with regard to the effect on respiratory complications of APDS.

The inhaled PI3Kδ inhibitor, GSK2269557 or Nemiralisib, is also currently being studied in APDS sponsored by GlaxoSmithKline (NCT02593539) (24, 26). Though an oral inhibitor maybe more effective for lymphoproliferation; it is proposed an inhaled inhibitor could benefit patients primarily affected by airway infection and bronchiectasis. The GSK2269557 clinical trial has not as yet reported results, but is described as a “multi-center, non-randomized, open-label, uncontrolled, single group study to investigate the safety and pharmacokinetics during 84 days repeat dosing treatment with 1,000 micrograms of inhaled in addition to standard of care, in subjects with APDS.” GSK2269557 is also currently being investigated as an anti-inflammatory treatment in Chronic Obstructive Pulmonary Disease (COPD) (26).

## Conclusion

The great clinical heterogeneity of APDS means that treatment needs to be carefully tailored to each patient's needs. Some asymptomatic family members need no treatment at all; patients with defective antibody production and recurrent infections may need supportive treatment such as prophylactic antibiotic and/or IRT. The decision as to which treatment needs to be made on clinical grounds. Patients with recurrent chest infection need very careful management in conjunction with a respiratory specialist as the development of life limiting and life threatening bronchiectasis is one of the major causes of early death in APDS. Sirolimus can be a very effective treatment for lymphoproliferative disease, but whether these benefits are sustained long term remains to be seen. Specific PI3Kδ inhibitor therapies appear very attractive and useful in resolving lymphoproliferation but serious side effects have been reported and the results from ongoing clinical trials will hopefully enable us to titrate the best dose and means of delivery as well as delineate long term risks and benefits. Preliminary data suggests that for younger patients in the process of developing life limiting and life threatening complications of APDS, HSCT is curative, but it carries a 10–20% mortality risk and it is unclear whether severe lung disease is reversed. Only with more outcome data from these therapies will the role of each of these treatment modalities become clear.

## Author contributions

All authors listed have made a substantial, direct and intellectual contribution to the work, and approved it for publication.

### Conflict of interest statement

The authors declare that the research was conducted in the absence of any commercial or financial relationships that could be construed as a potential conflict of interest.
